# Comparing Cortical Demyelination in Geriatric Mild Traumatic Brain Injury and Alzheimer’s Disease

**DOI:** 10.1093/geroni/igab046.2412

**Published:** 2021-12-17

**Authors:** Shania Wang, Nahian Chowdhury, Sean Mahoney, Andrei Irimia

**Affiliations:** 1 University of Southern California, Los Angeles, California, United States; 2 University of Southern California, University of Southern California, California, United States

## Abstract

Mild traumatic brain injury (mTBI) accelerates the rate of age-associated brain atrophy, whose pattern resembles the cortical neurodegeneration pattern observed in Alzheimer’s disease (AD). Because the ratio R of T1-to-T2-weighted magnetic resonance imaging (MRI) intensities is a surrogate measure of cortical myelin concentration, mapping and quantifying changes in this ratio can improve our understanding of demyelination after geriatric mTBI and AD. T1- and T2-weighted MRIs were acquired acutely and ~6 months post-injury from 68 healthy controls (HCs, age (years, y): μ = 76 y, σ = 4 y), 19 mTBIs (age μ = 70 y, σ = 5 y), and 33 ADs (age μ = 77, σ = 6). Volumes were co-registered using 3D Slicer’s BRAINSFit module, and T2-constrained segmentations of T1 volumes were obtained using FreeSurfer. R and its time changes were computed at each cortical location. When comparing mTBI and AD patients to HCs, significant differences in R were found across ~10% and ~23% of the cortex, respectively (p < 0.05). When comparing mTBI to AD, the former exhibited significantly less myelin content in the lateral, medial, and ventral temporal lobes (p < 0.05), on the medial aspects of superior parietal lobules and superior frontal gyri (p < 0.05), and in orbital gyri (p < 0.05), whereas AD subjects had less myelin content on lateral aspect of the parietal lobe (p < 0.05). These results highlight demyelination differences in mTBI and AD. Future studies should examine the long-term trajectories to quantify the risk of neurodegenerative disease after mTBI.

